# Measuring business-level expectations and uncertainty: survey evidence and the COVID-19 pandemic

**DOI:** 10.1007/s42973-021-00078-8

**Published:** 2021-07-07

**Authors:** Cheng Chen, Tatsuro Senga, Hongyong Zhang

**Affiliations:** 1 Clemson University, Clemson, USA; 2 Keio University, Tokyo, Japan; 3 Queen Mary University of London, London, UK; 4 RIETI, Tokyo, Japan

**Keywords:** Subjective uncertainty, Firm expectations, COVID-19, L2, M2, F1, D84

## Abstract

Utilizing a unique firm-level survey in Japan that contains five-bin forecasts for sales, we document three findings. First, firm-level subjective uncertainty is highly and positively related to volatility of past firm growth. Second, there are substantial variations in subjective uncertainty across firms, with a long right tail with extremely high subjective uncertainty. In addition, firms that have exposure to international businesses either through international trade or foreign direct investment have both higher average expected sales and subjective uncertainty. Finally, the sudden escalation of the COVID-19 pandemic in January–February 2020 led to a substantial increase in firms’ subjective uncertainty. Our triple-difference estimation results show that this effect is especially large for firms that have direct exposure to China through international trade and foreign direct investment.

## Introduction

A growing literature has highlighted the role of uncertainty shocks in slowing down business activities like hiring and investment. However, identifying uncertainty shocks and their causal relationship with economic activities remains an empirical challenge for at least two reasons. First, econometricians rarely observe firms’ subjective expectations about future outcomes directly, which makes it difficult to quantify the degree of subjective uncertainty faced by firms. Second, the causation may run in the opposite direction in that uncertainty may arise because of low economic activities. Omitted factors can also drive both uncertainty and economic activities.

This paper addresses such challenges by constructing a measure of subjective, firm-level uncertainty using the Business Plans and Expectations Survey (BPES), with its first wave in 2017 and its second wave in 2020 conducted by the Research Institute of Economy, Trade and Industry (RIETI).^1^ We elicit five-bin subjective probability distributions about future sales to construct a measure of firms’ expectations and uncertainty. With this measure, we investigate how business expectations and uncertainty changed after the initial outbreak of COVID-19 in China starting in January 2020 when the second wave of the survey was being collected. Exploiting heterogeneity among firms when each firm responded to the survey, we provide causal evidence in that the sudden outbreak of the COVID-19 pandemic in China increased firms’ subjective uncertainty, with the impact being more pronounced among firms that have transaction relationships with China.

As such, our empirical approach is guided by the timing of collecting survey responses during the outbreak of COVID-19 in China. The second wave of the survey started on January 7, 2020 and finished in mid-February. On January 23, Wuhan was locked down. On January 27, the Japanese government announced that COVID-19 was a designated infectious disease. We view this chain of events as information shocks in that firms that completed the survey before the week of January 20–26 had received little information concerning the COVID-19 pandemic, while firms that answered the survey after that week had received substantially more information concerning the COVID-19 pandemic. Moreover, firms that have a business relationship with China may be hit by such information shocks harder, because COVID-19 had not yet begun to affect the Japanese economy by mid-February when our survey ended. We directly test this issue by exploiting the panel nature of our dataset and implementing a triple difference, i.e., a difference-in-difference-in-differences (DDD) regression.

In particular, we divide firms that answered the survey in both 2017 and 2020 into two groups: those that answered the survey before January 23, 2020 and those that answered after January 23. We also divide firms into those that have a business relationship with China and those that do not. We define firms that answered our survey after the escalation of the COVID-19 pandemic *and* that have a business relationship with China as the treated firms. Our DDD estimation reveals that uncertainty increased among the treated firms from 2017 to 2020, compared with firms that answered the survey after the escalation of the COVID-19 pandemic *but* with no business relationship with China. Similarly, uncertainty increased among the treated firms from 2017 to 2020, relative to firms that have a business relationship with China *but* that answered the survey before the escalation of the COVID-19 pandemic. For sales expectations, we do not find such differential impacts among firms. These results are robust to controlling for firm fixed effects and can be further verified by a placebo test. In short, we conclude that the outbreak of the COVID-19 pandemic at its onset mainly triggered increased uncertainty instead of lowering firms’ expectations of sales growth.

There are three features of the case studied in the paper. First, given that the sudden escalation of COVID-19 was unlikely to be expected by Japanese firms, it was extremely unlikely that Japanese firms chose the date to answer the survey based on the situation of COVID-19. Therefore, we are able to provide *causal evidence* on effects of COVID-19 by comparing firms that answered the survey before the outbreak of COVID-19 with those that answered after the outbreak. For the same reason, it was extremely unlikely that Japanese firms changed their business relationships with China before the outbreak of COVID-19 by expecting that COVID-19 was coming. Consequently, we also compare firms that have business relationships with China with those that do not to provide *causal evidence* on the effects of COVID-19.

The second feature is that the Japanese economy was not hit by the COVID-19 pandemic until the end of our sample period. In January–February 2020, there were no closure requests or restriction of business activities in Japan. These policies were introduced in Japan from April 2020 and resulted in adverse impacts on firms’ sales as reported by Kawaguchi et al. (2021), who conducted a survey on Japanese small business managers’ expectations. Therefore, the effects we find are likely to be mainly driven by changes in information and expectations, rather than being driven by real demand/supply shocks.

The third feature is that our measures of expectations and uncertainty are obtained from the robust method that has been widely used across countries. Our method used here follows earlier surveys such as the Survey of Business Uncertainty (SBU) conducted by the Federal Reserve Bank of Atlanta and the Management and Organizational Practices Survey (MOPS) conducted by the US Census Bureau. Similar surveys have been conducted in the UK and Japan, including the Bank of England’s Decision Maker Panel (DMP), the Office for National Statistics’ Management and Expectations Survey, and the Japanese Managerial and Organizational Practices Survey (JP-MOPS), to elicit business expectations and investigate the impact of uncertainty on business performance. We validate our data by following the method adopted in these surveys to ensure comparability across countries.

Our paper contributes to the recent literature that studies how COVID-19 has impacted economic uncertainty. Based on the SUB and DMP, Baker et al. (2020) show that COVID-19-induced uncertainty rose rapidly in March 2020. Miyakawa et al. (2021) examine the impact of the COVID-19 pandemic, demonstrating that deteriorated expectations about their future sales growth contributed to firm exit observed in their data. Using aggregate data on Japanese multinational corporations (MNCs) in major foreign countries and regions, Zhang (2021) finds that COVID-19 had substantial impacts on the performance (sales, employment, and investment) of Japanese MNCs in Q1–Q3 of 2020. Due to the outbreak of COVID-19 in Q1 2020, total sales of Japanese multinational affiliates in China declined by $21.3\%$ year-on-year. At the same time, affiliates’ exports from China to Japan saw a decrease of $17.8\%$ year-on-year. The business confidence (as proxied by the diffusion index) of Japanese affiliates in China was also negatively affected by the COVID-19 pandemic in Q1 of 2020. As Baldwin and Tomiura (2020) point out, COVID-19 is contagious both economically and medically. As a result, the negative news and supply/demand shocks in China propagated to domestic firms in Japan through international linkages. A paper that is closely related to our paper is Morikawa (2021), which shows that the increase in firms’ subjective uncertainty is greater in the COVID-19 pandemic than in previous recessions.

The remainder of the paper is organized as follows. Section 2 briefly introduces the structure and items of the survey, and reports the validation and descriptive statistics of our data. Section 3 examines the impact of the COVID-19 pandemic on firm expectations and subjective uncertainty. Section 4 concludes.

## Survey

Our survey is a representative sample of Japanese firms above a certain threshold on size. In particular, we use the threshold adopted by the Basic Survey of Japanese Business Structure and Activities (henceforth Kikatsu data) collected by the Ministry of Economy, Trade and Industry (METI), which surveys firms that employ at least 50 employees and whose registered capital exceeds 30 million JPY. We focus on firms in manufacturing sectors and some service sectors (e.g., wholesale and retail trade, information services). As a result, in each wave, we end up with approximately 15,000 targeted firms from the Kikatsu data. In the survey form, we ask senior-level managers or CEOs to fill out the questionnaire. Participation in the survey was voluntary: $14.6\%$ (2185 firms) of the targeted firms responded to the first survey and $16.7\%$ (2641 firms) to the second. The data we obtained included $45\%$ manufacturing firms and $55\%$ service firms.

The main part of the survey asked firms to report their forecasts for both aggregate-level and firm-level economic variables. Specifically, the firm was asked to report a distribution of their forecasts (i.e., five bins) for the exchange rate, the GDP growth rate, and firm sales for the fiscal years 2018 and 2020 (i.e., 1 year ahead). As shown in Table 1, in our second survey, we asked about expected sales by the end of fiscal year 2019 (i.e., March), five scenarios related to forecast in fiscal year 2020, and the expected probability of each scenario. This type of survey has also been conducted in the US and UK. The second time the survey was collected was in January–February 2020, when the COVID-19 outbreak had already begun to spread in China. This made it possible to analyze how an unforeseen event affects firms’ future outlook.

**Table 1:**
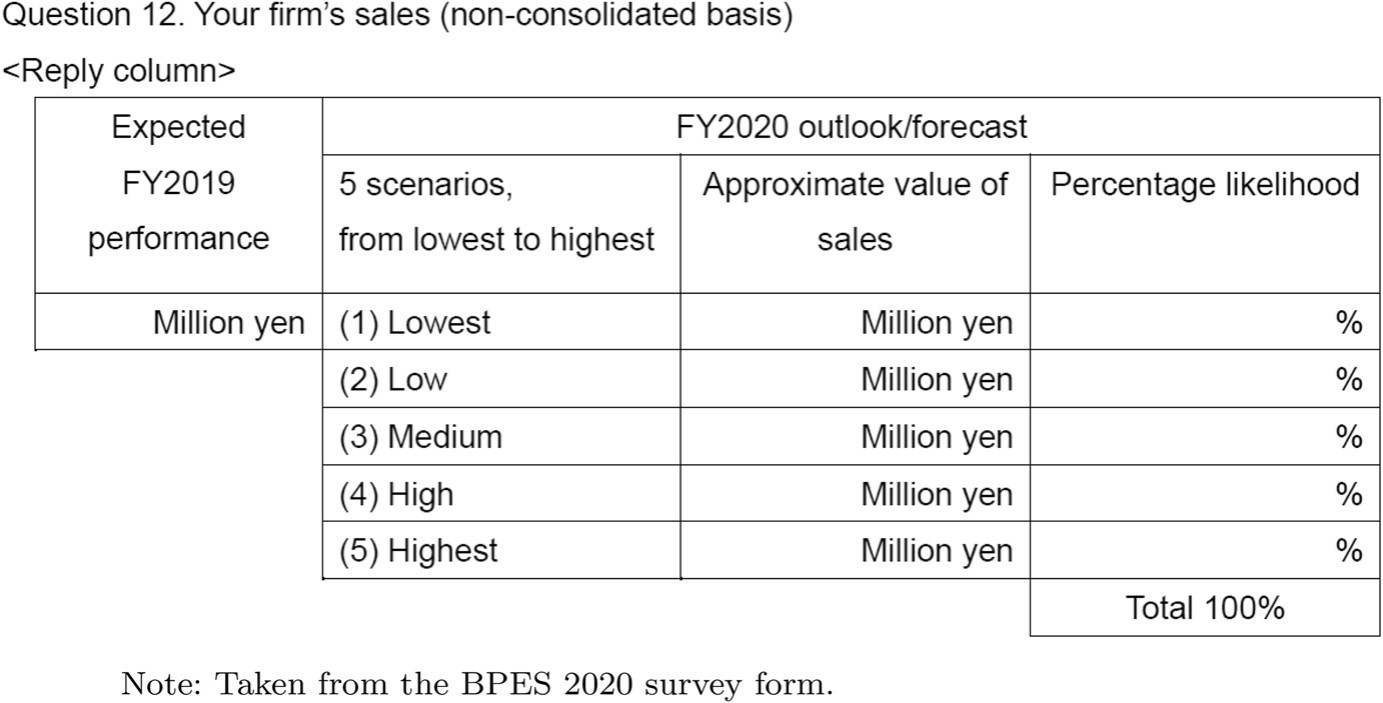
Survey items on firm sales forecasts and probability

Following Altig et al. (2020), we assume that the future sales growth rates of a firm are in a discrete probability distribution. Suppose the distribution has $N (i=1,2,3,4,5)$ support points. The future sales growth rate values $\mathrm{SalesGR}_i$ (with associated probabilities $p_i$) can be defined as

$$\begin{aligned} \mathrm{SalesGR}_{i,t+1}=\frac{\mathrm{sales\;fore}_{i,t+1}-\mathrm{sales}_{t}}{\left( \frac{\mathrm{sales\;fore}_{i,t+1}+\mathrm{sales}_{t}}{2}\right) }, \end{aligned}$$

where the denominator is a simple average of realized sales in the current fiscal year, $\mathrm{sales}_{t}$, and sales forecast for the next fiscal year, $\mathrm{sales\;fore}_{i,t+1}$. The variance in future outlook can be calculated from responses obtained to construct a firm-level uncertainty index. Specifically, we calculate the firm’s mean forecast of the sales growth rate for year $t+1$ as

$$\begin{aligned} \mathrm{Mean(SalesGR)}_{t+1} = \sum _{i=1}^N p_i \cdot \mathrm{SalesGR}_{i,t+1}, \end{aligned}$$

and its subjective uncertainty as the standard deviation of the expected sales growth rate

$$\begin{aligned} \mathrm{SD(SalesGR)}_{t+1} = \left[ \sum _{i=1}^N p_i (\mathrm{SalesGR}_{i,t+1} - \mathrm{Mean(SalesGR)}_{t+1})^2\right] ^{\frac{1}{2}}. \end{aligned}$$

Alternatively, we can calculate the coefficient of variation (CV) of the sales forecasts (in levels):

$$\begin{aligned} \mathrm{CV(Sales)}_{t+1}=\frac{\mathrm{SD(Sales\;fore)}_{t+1}}{\mathrm{Mean(Sales\;fore)}_{t+1}}, \end{aligned}$$

where $\mathrm{SD(Sales\;fore)}_{t+1}$ and $\mathrm{Mean(Sales\;fore)}_{t+1}$ are the standard deviation and mean of the five-bin sales forecasts (i.e., levels) for year $t+1$.

### Validation of data

As it is rare for firms to report the distribution of their forecasts for future economic outcomes, we first show that these data provide valid information for our following analysis. We follow Bloom et al. (2020) to implement several validation checks for our data and focus on firms that reported the distribution of forecasts. As the forecasts for firm sales are the key variable in our analysis, we provide the validation tests for this variable.^2^

We implement four checks. First, we calculate the number of observations whose reported probabilities for the five bins do not add up to one.^3^ Second, we calculate the number of observations whose reported forecasts do not weakly increase with the index of the bins.^4^ Third, we calculate the number of observations that report the same forecast in two different bins.^5^ Finally, we calculate the number of observations that have a point mass in one of the five forecasts (i.e., $100\%$ for one forecasted value). Table 2 shows that only a small number of firms failed to satisfy the required criteria. Therefore, we believe that most responses to our questions on distributional forecasts are reliable. As our sample size is already relatively small, we adjust the data by correcting some of the detected mistakes and use the whole sample for the following analysis.^6^ We also conduct the same checks for the survey sample in 2020 and the results are quite similar.

**Table 2:** Validation of data (2017)

	Yes	No
Probabilities add up to $100\%$?	1597	64
Forecasts weakly increase with the index of the bins?	2165	20
Same forecast in two different bins?	25	2160
One forecast has the point mass (i.e., $100\%$)?	66	2119

Notes: Total number of observations is 2185. There are roughly 500 firms that did not report the distribution of their forecasts. Other than row one, we include these observations into the calculation of summary statistics and treat them as firms that satisfy all the criteria

**Fig. 1:**
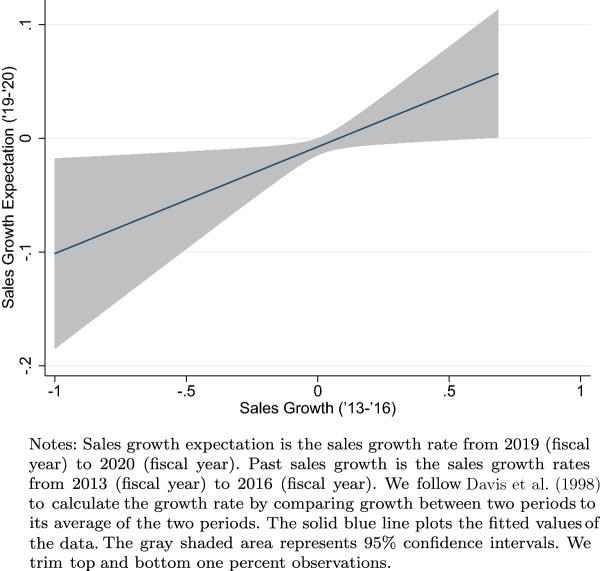
Past and forecasted sales growth

**Fig. 2:**
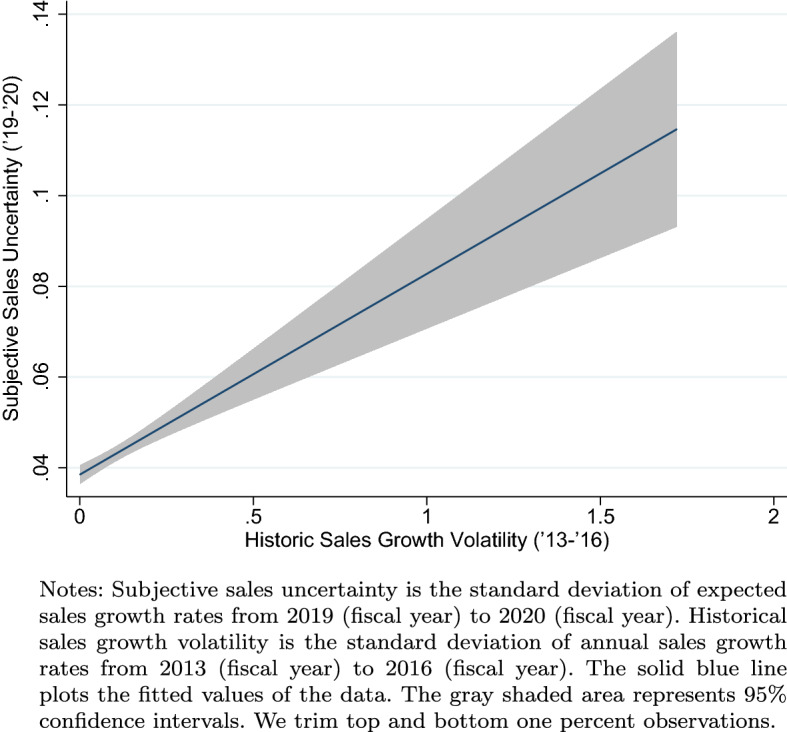
Subjective uncertainty and sales volatility

Another type of check we implement is to show the positive relationship between the forecasted sales growth and two objective measures of firm growth rate and volatility. First, Fig. 1 shows a highly positive correlation between past sales growth and forecasted sales growth.^7^ Next, Fig. 2 indicates that the subjective uncertainty measure we construct is highly positively correlated with the historical sales growth volatility, which is an objective measure of firm-level uncertainty. Together, the two figures show that firms were probably making their forecasts rationally and reasonably, and this evidence is similar to the empirical patterns documented in Bloom et al. (2020) for US firms.

### Descriptive statistics

We present some descriptive statistics that highlight some key patterns in the data. Table 3 presents summary statistics of forecasted sales growth rate, sales growth probability, and calculated sales uncertainty. The mean sales growth rates of the five scenarios, from lowest to highest, are in weakly increasing order. The sum of the mean sales growth probabilities of the five scenarios is $100\%$. The mean expected sales growth is close to zero, with a standard deviation of 0.138. The mean sales uncertainty is 0.044 and the standard deviation is 0.033.

**Table 3:** Descriptive statistics (2017 and 2020)

	Count	Mean	SD	p5	p25	p50	p75	p95
Sales growth rate 1	3339	− 0.092	0.193	− 0.296	− 0.125	− 0.064	− 0.023	0.043
Sales growth rate 2	3323	− 0.042	0.165	− 0.199	− 0.069	− 0.028	0.000	0.087
Sales growth rate 3	3339	0.008	0.147	− 0.118	− 0.015	0.000	0.033	0.149
Sales growth rate 4	3307	0.048	0.154	− 0.069	0.006	0.032	0.074	0.208
Sales growth rate 5	3320	0.086	0.167	− 0.023	0.026	0.063	0.120	0.279
Sales growth prob. 1 (%)	3429	10.686	7.849	2.000	5.000	10.000	10.000	20.000
Sales growth prob. 2 (%)	3429	18.535	10.145	5.000	10.000	20.000	20.000	35.000
Sales growth prob. 3 (%)	3429	43.200	16.468	20.000	30.000	40.000	50.000	70.000
Sales growth prob. 4 (%)	3429	17.850	10.287	3.000	10.000	20.000	20.000	30.000
Sales growth prob. 5 (%)	3429	9.733	7.162	0.000	5.000	10.000	10.000	20.000
Expected growth rate	3287	0.004	0.138	− 0.129	− 0.023	− 0.000	0.032	0.139
Expected sales (log)	3292	8.363	1.219	6.492	7.511	8.319	9.128	10.479
Sales uncertainty (CV)	3290	0.044	0.033	0.009	0.022	0.036	0.058	0.109
Sales uncertainty (SD)	3223	0.044	0.033	0.009	0.021	0.036	0.058	0.108

Notes: Sales uncertainty (SD) is the subjective uncertainty defined as the standard deviation of the expected sales growth rate, sales uncertainty (CV) is the subjective uncertainty defined as the coefficient of variation of the sales forecasts

**Fig. 3:**
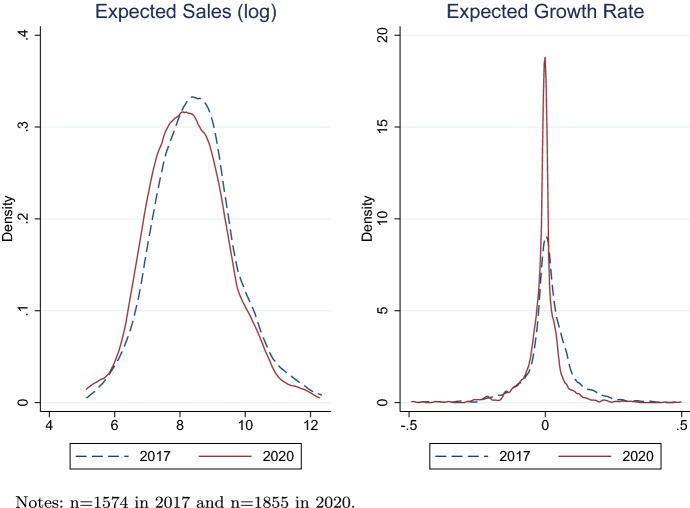
Expected sales

Figure 3 shows the distributions of log average expected sales (left panel) and average expected growth rates (right panel) in 2017 and 2020 samples. Relative to 2017, both expected sales and growth rates are lower in 2020. Probably because the real GDP growth rate of Japan was only $0.3\%$ in 2019 (it was much higher at $1.7\%$ in 2017), firms tended to have lower forecasts on sales growth rates in 2020. We conduct a two-sample Kolmogorov–Smirnov (KS) test to examine if there are any differences in the distribution for these two groups. The results show that the distributions of expected sales in 2017 and 2020 in each panel are not equal, with statistical significance at the $1\%$ level.

**Fig. 4:**
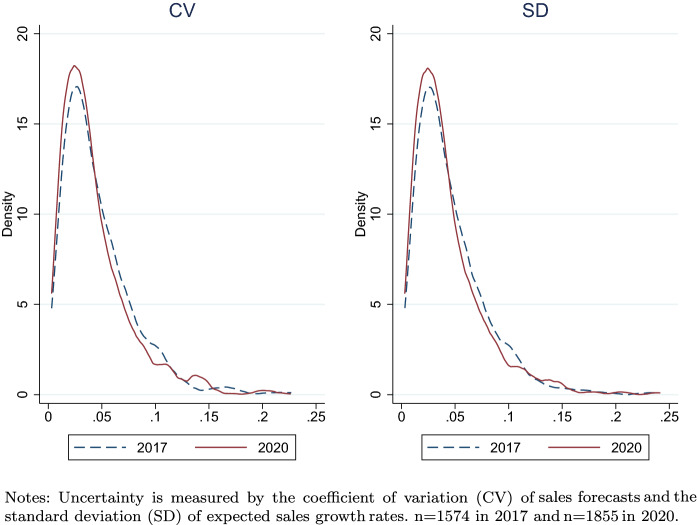
Sales uncertainty

Figure 4 plots the kernel density of sales uncertainty in terms of the coefficient of variation (CV) of sales forecasts (left panel) and the standard deviation (SD) of expected sales growth rates (right panel). It is clear that there are substantial variations in subjective uncertainty across firms, with a long right tail. The distributions in 2017 and 2020 are quite similar, which shows that the samples from the 2 years are comparable and thus suitable for panel regressions. Furthermore, the sales uncertainty distribution in 2017 is—slightly but visibly—to the right in both panels, showing that the subjective uncertainty was higher in the 2017 survey sample.^8^ To examine the underlying factors of the uncertainty faced by firms, the survey also asked the respondents to cite items that affect their forecasted business and operating environments (multiple answers accepted). Of the surveyed firms in 2017, $60\%$ cited Japan’s economic growth rate, compared with $49\%$ for the domestic price level and $35\%$ for the economic policies of the government and the Bank of Japan (BOJ). Of the policies of the government and the BOJ, the tax policy (e.g., corporate tax, consumption tax, etc.) was cited by $73\%$, the policy for the system concerning the labor standard and supervision was cited by $48\%$, and monetary policy was cited by $36\%$.

**Fig. 5:**
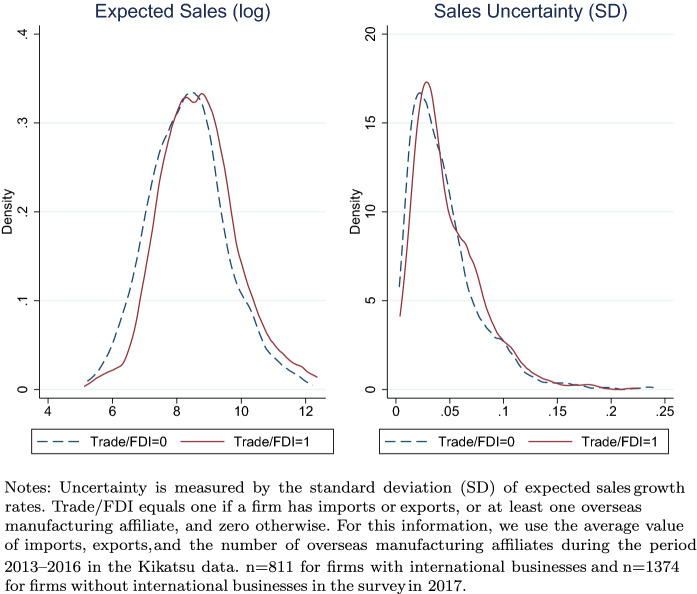
Expected sales and sales uncertainty: international business

The results of the survey also indicate the possibility that firm-level uncertainty was related to international trade and foreign direct investment (FDI). Figure 5 plots the kernel density of expected sales growth rates and sales uncertainty by firms with international businesses and firms without international businesses in the survey in 2017. International businesses can be in the form of importing/exporting and/or FDI. Interestingly, compared with firms without international businesses, firms with international businesses tended to have larger average expected sales, but also higher sales uncertainty. The differences in the distributions between these two groups in both panels are confirmed by the KS test. Related to this finding, the survey also asked firms about the factors in major foreign countries (e.g., China, the US) that would affect the degree of uncertainty about their business plans. Firms could choose multiple factors among economic policy, trade policy, geopolitical risks, and others by major country. For example, in our second survey, about one-quarter of firms regarded China’s economic policy and trade policy (tariff hikes induced by the US–China trade war that started from March 2018) as the most important factors affecting their business plans. Moreover, $15.7\%$ of firms answered that the tightness of the local market in China affected the degree of uncertainty concerning their business plans.

## Impact of COVID-19

### Background and empirical strategy

We first briefly introduce the background of the outbreak of COVID-19 in January–February 2020. On December 31, 2019, the Wuhan Municipal Health Commission released the first public message about early signs of a pneumonia outbreak in the city. On January 1, 2020, the Huanan Seafood Market was closed for cleaning and disinfection. The health authorities in Wuhan reported 44 cases on January 3. On January 9, the World Health Organization (WHO) announced that Chinese authorities had determined that the outbreak was caused by a novel coronavirus. On January 15, the Japanese Ministry of Health, Labor and Welfare reported a confirmed case in a person who had traveled to Wuhan. This was the second confirmed case detected outside of China. On January 20, the China National Health Commission reported that the virus was human-to-human transmissible. Wuhan was locked down on January 23. The next day, Tokyo confirmed its first case of COVID-19 and the Japanese government announced that it would provide repatriation services for all Japanese citizens in Hubei Province on the same day. On January 27, the Abe administration designated the COVID-19 as an “infectious disease” under the Infectious Diseases Control Law. The WHO declared the COVID-19 outbreak a Public Health Emergency of International Concern on January 30 (and a pandemic on March 11). On February 1, the Japanese government enacted restrictions to deny entry to foreign citizens who had visited Hubei province within 14 days and to those with a Chinese passport issued from there. On February 13, a woman died in Kanagawa Prefecture, marking the first death from COVID-19 in Japan and the third death outside mainland China. It is worth noting that our second survey that started from January 7, 2020 and ended on February 18, 2020 accidentally coincided with the outbreak of COVID-19. Until February 18, 2020, the number of confirmed cases in China was 74,185 compared with only 74 in Japan.^9^

**Fig. 6:**
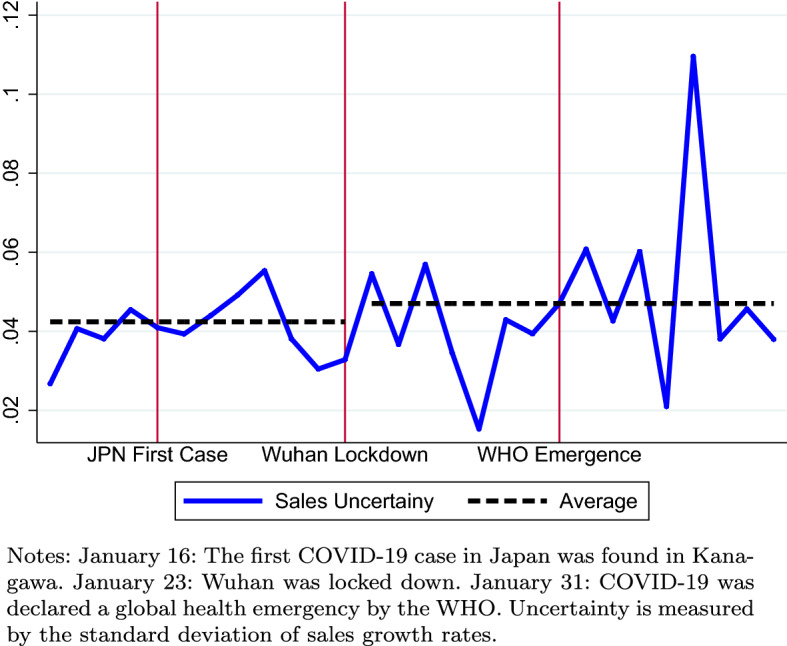
COVID-19 and subjective uncertainty

To examine the impact of COVID-19 on firm expectations and uncertainty, we exploit the panel nature of our dataset and implement a triple-difference, i.e., difference-in-difference-in-differences (DDD) regression. Given the unexpectedness of the escalation of COVID-19, we believe that there was an informational shock that occurred around January 23, 2020 (i.e., in the week of January 20–26) when Wuhan was locked down, and which persisted until the end of the survey period. In the empirical analysis, we divide firms that answered the survey in both 2017 and 2020 into two groups: those that answered the survey before January 23, 2020 when the severity of COVID 19 pandemic was substantially elevated (due to the lockdown of Wuhan) and those that answered after January 23. The rationale is that firms that completed the survey before January 23 had received little information concerning the COVID-19 pandemic, while firms that answered the survey after this date had received substantially more information concerning the COVID-19 pandemic. Figure 6 plots the daily subjective uncertainty of the firms that responded to our second survey. It is clear that the average sales uncertainty increased after the lockdown of Wuhan (January 23), although the daily data are very volatile due to numerous macroeconomic and firm-specific shocks. We then compare how firms in the first group changed their expected sales from 2017 to 2020 *relative to* firms in the second group. Moreover, we also look at how the second moment of sales expectations (i.e., subjective uncertainty) changed from 2017 to 2020 for the firms in the first group (relative to firms in the second group). Our main finding is that firms that answered the survey after the sudden escalation of the COVID-19 pandemic had lowered their (average) expected sales and increased their subjective uncertainty relative to those that answered the survey before the sudden escalation of the COVID-19 pandemic, although the estimated impacts are marginally (statistically) significant.

Relatedly, we also exploit the fact that the escalation of the COVID-19 pandemic occurred mainly inside China (at least before mid-February) in our identification strategy. Specifically, we first divide firms into four groups: those that answered the survey before or after the week of January 20–26, and whether the firm has a business relationship with China or not. We treat the group of firms that answered the survey after the escalation of COVID-19 *and* with a business relationship with China (either via imports/exports or by having production bases in China) as the treatment group. We then implement a DDD estimation and find that the treated firms increased their subjective uncertainty from 2017 to 2020 compared with firms that answered the survey after the escalation of COVID-19 *but* with no business relationship with China. Similarly, the treated firms increased their subjective uncertainty from 2017 to 2020 relative to firms with a business relationship with China *but* that answered the survey before the escalation of COVID-19. The estimated differential impact on firms that have a business relationship with China is both statistically and quantitatively significant. However, we do not find this differential impact on firms that have a business relationship with China when using the average sales expectation as the outcome variable. Overall, our evidence supports the argument that the sudden escalation of the COVID-19 pandemic *in its early days* mainly increased uncertainty *faced by firms outside China*.

There are several interesting features about studying Japanese firms during this period. First, the survey period was from January 9 to February 18, 2020, which covers the sudden escalation of the COVID-19 pandemic, namely the week of January 20–26. As a result, we are able to implement the difference-in-differences (DID) and the DDD analyses. Second, although the sudden escalation of the COVID-19 pandemic occurred in the week of January 20–26, there were very few cases in Japan up until the end of our sample period.^10^ Moreover, because the Japanese economy had not been hit by the COVID-19 pandemic before the end of our sample period, the effects that we find are most likely driven by changes in information and expectations rather than by real demand/supply shocks.^11^ Third, as Japan has a very close economic relationship with China, the outbreak of COVID-19 in China likely affected the business plans and expectations of Japanese firms. Finally, our sample covers a wide range of sectors including both manufacturing and services and a wide range of regions (i.e., prefectures).

We estimate the following empirical equations:

1 $$\begin{aligned} y_\mathrm{it}= & {} \beta _0+ \beta _1D(\mathrm{year}=2020)+ \beta _2D(\mathrm{year}=2020)\times D(\mathrm{date}>\mathrm{Jan.}/26)\nonumber \\&+\beta _3D(\mathrm{year}=2020)\times \mathrm{China}_{i}+\beta _4D(\mathrm{year}=2020)\times D(\mathrm{date}>\mathrm{Jan.}/26)\nonumber \\&\times \mathrm{China}_{i}+\beta _5\mathrm{log(age)}_{i}+ \mathrm{FE}_{i}+ \epsilon _\mathrm{it}, \end{aligned}$$

2 $$\begin{aligned} y_\mathrm{it}= & {} \delta _0+ \delta _1D(\mathrm{year}=2020)+\delta _2D(\mathrm{year}=2020)\times D(\mathrm{date}>\mathrm{Jan.}/22)\nonumber \\&+\delta _3D(\mathrm{year}=2020)\times \mathrm{China}_{i}+ \delta _4D(\mathrm{year}=2020)\times D(\mathrm{date}>\mathrm{Jan.}/22)\nonumber \\&\times \mathrm{China}_{i}+\delta _5\mathrm{log(age)}_{i}+ \mathrm{FE}_{i}+ \epsilon _\mathrm{it}, \end{aligned}$$

where *i* and *t* denote firm and year, respectively. $y_\mathrm{it}$ is the logarithm of average expected sales, the coefficient of variation of the five-bin distribution of expected sales, and the standard deviation of expected sales growth rates (across five bins). *D* denotes year or date dummy and FE denotes fixed effect. China is a dummy variable that equals one if the firm has (1) imports from China, or (2) exports to China, or (3) at least one manufacturing affiliate in China, and zero otherwise. For this information, we use the average value of (1) imports from China, (2) exports to China, and (3) the number of manufacturing affiliates in China during the period 2013–2016 in the Kikatsu data. We have 161 firms that have business relationships with China.^12^

Before presenting the empirical results, we emphasize some details of our empirical specifications. First, there are three main events that substantially escalated the severity of the COVID-19 pandemic. On January 20, 2020, human-to-human transmission was confirmed in China and a large wave of broadcasting COVID-19 started to appear. On January 23, the lockdown in Wuhan began. On January 27, the Japanese government announced that COVID-19 was a designated infectious disease. Therefore, we set the cutoff date of the escalation as January 23 (or January 27). Next, roughly $62\%$ of firms in our sample in 2020 mailed their answers to TDB,^13^ and there is probably a time lag between the time when managers filled out the survey and the time when the completed survey arrived at TDB via post. We exclude firms that answered the survey between January 20 and January 22 (or January 26) in our regressions, as we want to exclude observations whose dates of completion of the survey are either before or after knowing about COVID-19. Third, we examine the heterogeneous effect across different firms by looking at how firm-specific exposure to the Chinese economy affects changes in firms’ expectations after the escalation of COVID-19. The hypothesis is that Japanese firms with direct exposure to the Chinese economy are likely to be affected most by COVID-19. Fourth, as we always include firm fixed effects into the regressions, we only use observations with completed surveys in both 2017 and 2020 in the regressions, which reduces our sample size substantially.^14^ Finally, we trim out average expected sales and subjective uncertainty of firms’ sales expectations from both below and above the $1\%$ level, as some numbers of sales expectations are very likely to be outliers. The summary statistics of the variables are reported in Appendix Table 9.^15^

### Main results

We first investigate how the escalation of COVID-19 affected the first moment of firms’ sales expectations in Table 4 using the logarithm of the expected sales as the dependent variable. We implement DID regressions with the category of firms that answered the survey after January 26 (or January 22) as our treatment group, and the results are reported in columns (1) and (2). Specifically, we regress the average expected sales on a year dummy variable, $1(\mathrm{year}=2020)$, and an interaction term between this variable and whether or not the firm answered the survey after January 26 (or January 22). In addition, we control for firm age and include firm fixed effects into the regressions.

We find that firms that answered the survey after the escalation of the COVID-19 pandemic reduced their average expected sales from 2017 to 2020 relative to firms that answered before the escalation, although the estimated effect is only marginally significant. Next, we examine the heterogeneous effect on firms that have (or do not have) exposure to the Chinese economy, as the initial outbreak of COVID-19 was mainly restricted to China. Specifically, we introduce a dummy variable (*China*) that equals one when the firm has a business relationship with China, either via trade or by having production facilities in China. We then run a DDD regression by interacting the year dummy variable with both the *China* dummy variable and whether the firm answered the survey after January 26 (or January 22). We also include two double-interaction terms: (1) the year dummy variable interacted with the *China* dummy variable, and (2) the year dummy variable interacted with whether the firm answered the survey after January 26 (or January 22) to control for differential time trends for firms that have (or do not have) exposure to the Chinese economy and firms with different sizes.^16^ Columns (3) and (4) of Table 4 do not show different impacts on firms that have (or do not have) exposure to the Chinese economy. In sum, we conclude that the outbreak of COVID-19 did not seem to make firms outside China pessimistic about their future sales, at least at its outset.

**Table 4:** Expected sales and COVID-19

	(1)	(2)	(3)	(4)
	$$\mathrm{Sales}_\mathrm{avg}$$			
$1(\mathrm{year}=2020)$	− 0.039 (0.071)	− 0.030 (0.065)	− 0.031 (0.080)	− 0.022 (0.075)
$1(\mathrm{year}=2020)\times 1(\mathrm{date}>\mathrm{Jan.}/26)$	− 0.105$^{+}$ (0.073)		− 0.141^+^ (0.092)	
$1(\mathrm{year}=2020)\times 1(\mathrm{date}>\mathrm{Jan.}/22)$		− 0.115^+^ (0.070)		− 0.155* (0.090)
$1(\mathrm{year}=2020)\times \mathrm{China}$			− 0.039 (0.100)	− 0.040 (0.100)
$1(\mathrm{year}=2020)\times 1(\mathrm{date}>\mathrm{Jan.}/26)\times \mathrm{China}$			0.117 (0.144)	
$1(\mathrm{year}=2020)\times 1(\mathrm{date}>\mathrm{Jan.}/22)\times \mathrm{China}$				0.129 (0.134)
log(firm age)	1.740* (0.904)	1.600** (0.765)	1.776* (0.909)	1.632** (0.776)
Firm FE	Yes	Yes	Yes	Yes
$N$	648	736	648	736
$R^{2}$	0.916	0.911	0.916	0.911

Notes: $\mathrm{Sales}_\mathrm{avg}$ is the log average expected sales for the next calendar year. Standard errors are clustered at the firm level. Dependent variable is trimmed out from both below and above at $1\%$ level. Firms that answered our survey between Jan./20/2020 and Jan./26/2020 are excluded from columns 1 and 3. Firms that answered our survey between Jan./20/2020 and Jan./22/2020 are excluded from columns 2 and 4. Significance levels: $^+$0.20, *0.10, **0.05, ***0.01

The impact of COVID-19 on firm-level uncertainty (i.e., the second moment of sales expectations distribution) is starkly different from its impact on the first moment of sales expectations. In Tables 5 and 6, we regress two measures of firm-level uncertainty: the coefficient of variation of the five-bin distribution of expected sales and the standard deviation of expected sales growth rates (across five bins), respectively, on the same set of explanatory variables used to generate Table 4. The coefficients reported in columns (1) and (2) of the two tables show that firm-level uncertainty increased by 0.007 after the escalation of COVID-19, with statistical significance at the $10\%$ level in Table 6. This increase is roughly $16\%$ of the average firm-level uncertainty in our sample. Importantly, our DDD estimation results reported in columns (3) and (4) of the two tables show that the triple-interaction terms are positive and statistically significant, and the quantitative magnitudes are large. For instance, column (3) of Table 5 implies that firms that answered the survey after the escalation of COVID-19 *and* that have a business relationship with China increased their sales uncertainty by 0.020 (from 2017 to 2020) compared with firms that answered the survey after the escalation of COVID-19 *but* with no business relationship with China. This increase is roughly $45\%$ of the average firm-level uncertainty in our sample. Overall, our empirical analysis shows that the sudden escalation of the COVID-19 pandemic increased the second moment of firms’ sales expectations. The quantitative magnitudes are large, which substantiates the importance of receiving new information in shaping firm-level uncertainty.

**Table 5:** Sales uncertainty (CV) and COVID-19

	(1)	(2)	(3)	(4)
	$$\mathrm{Sales}_\mathrm{cv}$$			
$1(\mathrm{year}=2020)$	− 0.002 (0.004)	− 0.003 (0.004)	− 0.002 (0.005)	− 0.003 (0.005)
$1(\mathrm{year}=2020)\times 1(\mathrm{date}>\mathrm{Jan.}/26)$	0.007$^{+}$ (0.004)		0.000 (0.005)	
$1(\mathrm{year}=2020)\times 1(\mathrm{date}>\mathrm{Jan.}/22)$		0.006$^{+}$ (0.004)		0.001 (0.005)
$1(\mathrm{year}=2020)\times \mathrm{China}$			− 0.002 (0.006)	− 0.002 (0.006)
$1(\mathrm{year}=2020)\times 1(\mathrm{date}>\mathrm{Jan.}/26)\times \mathrm{China}$			0.020** (0.010)	
$1(\mathrm{year}=2020)\times 1(\mathrm{date}>\mathrm{Jan.}/22)\times \mathrm{China}$				0.016* (0.009)
log(firm age)	− 0.037 (0.050)	− 0.029 (0.046)	− 0.026 (0.046)	− 0.023 (0.045)
Firm FE	Yes	Yes	Yes	Yes
$N$	636	724	636	724
$R^{2}$	0.674	0.679	0.682	0.684

Notes: $\mathrm{Sales}_\mathrm{cv}$ is the coefficient of variation of the (five-bin) expected sales distribution. Standard errors are clustered at firm level. Dependent variable is trimmed out from both below and above at $1\%$ level. Firms that answered our survey between Jan./20/2020 and Jan./26/2020 are excluded from columns 1 and 3. Firms that answered our survey between Jan./20/2020 and Jan./22/2020 are excluded from columns 2 and 4. Significance levels: $^+$0.20, *0.10, **0.05, ***0.01

**Table 6:** Sales uncertainty (SD) and COVID-19

	(1)	(2)	(3)	(4)
	$$\mathrm{Sales}^\mathrm{pc}_\mathrm{sd}$$			
$1(\mathrm{year}=2020)$	− 0.002 (0.005)	− 0.003 (0.004)	− 0.002 (0.005)	− 0.003 (0.005)
$1(\mathrm{year}=2020)\times$ $1(\mathrm{date}>\mathrm{Jan.}/26)$	0.007* (0.004)		0.002 (0.005)	
$1(\mathrm{year}=2020)\times$ $1(\mathrm{date}>\mathrm{Jan.}/22)$		0.007* (0.004)		0.002 (0.005)
$1(\mathrm{year}=2020)\times$ China			− 0.002 (0.006)	− 0.002 (0.006)
$1(\mathrm{year}=2020)\times$ $1(\mathrm{date}>\mathrm{Jan.}/26)$ × China			0.017* (0.010)	
$1(\mathrm{year}=2020)\times$ $1(\mathrm{date}>\mathrm{Jan.}/22)$ × China				0.014^+^ (0.009)
log(firm age)	− 0.041 (0.051)	− 0.033 (0.047)	− 0.032 (0.048)	− 0.028 (0.046)
Firm FE	Yes	Yes	Yes	Yes
$N$	612	694	612	694
$R^{2}$	0.689	0.692	0.695	0.696

Notes: $\mathrm{Sales}^\mathrm{pc}_\mathrm{sd}$ is the standard deviation of expected sales growth rates (across five bins). Standard errors are clustered at the firm level. Dependent variable is trimmed out from both below and above at $1\%$ level. Firms that answered our survey between Jan./20/2020 and Jan./26/2020 are excluded from columns 1 and 3. Firms that answered our survey between Jan./20/2020 and Jan./22/2020 are excluded from columns 2 and 4. Significance levels: $^+$0.20, *0.10, **0.05, ***0.01

### Placebo test

In our DDD specifications, we carefully include the interaction term between the China dummy and the year 2020 dummy to control for various China-specific shocks to disentangle the impact of the COVID-19 shock. This is crucial because the escalation of the US–China trade war may affect the aggregate Chinese economy and the slowdown of China’s economic growth, which may particularly affect the expectations and subjective uncertainty of Japanese firms that have business relationships with China. Therefore, if we omit this variable, the estimated effect of our triple-interaction terms will not only reflect the effect of the COVID-19 shock, but will also reflect the overall effects of the trade war and increases in economic and geopolitical uncertainties in China.

There may be a concern that, even after controlling for the interaction term between the China dummy and the year 2020 dummy, our triple-interaction term may still pick up some effects of other shocks (e.g., exchange rates) or firm heterogeneity (e.g., firms engaging in international trade and investment tend to have high subjective uncertainty as shown in Fig. 5). Here, we perform a placebo test to show that, after the sudden escalation of COVID-19, the triple-interaction term with a China dummy turns out to have predictive power, while the triple-interaction term with a *non-China* dummy does not matter. Specifically, we look at firms’ international business relationships with non-China countries and regions. We define a non-China dummy variable that equals one if the firm has no business relationship with China but has (1) imports from non-China countries, or (2) exports to non-China countries, or (3) at least one manufacturing affiliate in a non-China country, and zero otherwise.^17^ We have 69 firms that have a business relationship with non-China countries but no business relationship with China at the same time. We see the sudden escalation of COVID-19 in January–February 2020 as a negative news shock for Japanese firms that engage in trade with China and/or investment in China, but there is no evidence that such a news shock was related to firms that have international business linkages with non-China countries and regions.

We conduct a placebo test with triple interactions with a non-China dummy. The regression results are reported in Table 7. Using the same set of controls specified previously, column (4) shows that the triple-interaction term with a non-China dummy has no predictive power after the sudden escalation of COVID-19, while column (3) shows that the sales uncertainty even reduced somewhat. In sum, the placebo test shows that the triple-interaction term with the China dummy captures the impact of the sudden escalation of COVID-19 well.

**Table 7:** Placebo test: business relationships with non-China

	(1)	(2)	(3)	(4)
	$$\mathrm{Sales}_\mathrm{cv}$$			
$1(\mathrm{year}=2020)$	− 0.002 (0.004)	− 0.003 (0.004)	− 0.002 (0.005)	− 0.003 (0.005)
$1(\mathrm{year}=2020)\times 1(\mathrm{date}>\mathrm{Jan.}/26)$	0.007$^{+}$ (0.004)		0.008$^{+}$ (0.005)	
$1(\mathrm{year}=2020)\times 1(\mathrm{date}>\mathrm{Jan.}/22)$		0.006$^{+}$ (0.004)		0.007$^{+}$ (0.004)
$1(\mathrm{year}=2020)\times {\text {non-China}}$			0.001 (0.006)	0.001 (0.006)
$1(\mathrm{year}=2020)\times 1(\mathrm{date}>\mathrm{Jan.}/26)\times {\text {non-China}}$			− 0.010$^{+}$ (0.007)	
$1(\mathrm{year}=2020)\times 1(\mathrm{date}>\mathrm{Jan.}/22)\times {\text {non-China}}$				− 0.005 (0.008)
log(firm age)	− 0.037 (0.050)	− 0.029 (0.046)	− 0.038 (0.050)	− 0.030 (0.046)
Firm FE	Yes	Yes	Yes	Yes
$N$	636	724	636	724
$R^{2}$	0.674	0.678	0.675	0.678

Notes: $\mathrm{Sales}_\mathrm{cv}$ is the coefficient of variation of the (five-bin) expected sales distribution. Standard errors are clustered at the firm level. Dependent variable is trimmed out from both below and above at $1\%$ level. Firms that answered our survey between Jan./20/2020 and Jan./26/2020 are excluded from columns 1 and 3. Firms that answered our survey between Jan./20/2020 and Jan./22/2020 are excluded from columns 2 and 4. Significance levels: $^+$0.20, *0.10, **0.05, ***0.01

### Mechanism

In previous sections, we identified a significant and positive impact of the escalation of the COVID-19 pandemic on firms’ subjective uncertainty, especially for firms that have direct exposure to China through international linkages. In this subsection, we provide further evidence to shed light on the underlying channels through which the COVID-19 shock affects firms’ subjective uncertainty. Specifically, to measure the exposure of firms’ business activities to China, we construct three continuous variables: (1) the share of imports from China in total value of sourcing; (2) the share of exports to China in total sales; and (3) the number of manufacturing affiliates in China. We use the average values of import share, export share, and the number of manufacturing affiliates, respectively, during the period 2013–2016 in the Kikatsu data. Using these continuous measures instead of a China dummy, we conduct additional DDD estimations. The results are reported in Table 8.

In column (1), we use the triple-interaction term with the share of imports from China and find that firms that answered the survey after the escalation of COVID-19 and rely more on imported inputs from China increased their sales uncertainty (CV) by 0.69 (average import share: 0.034) compared with firms that answered the survey after the escalation of COVID-19 but that have low/no reliance on Chinese imports. The magnitude is found to be economically and statistically significant. Given that the sales uncertainty of firms that have a business relationship with China increased by 0.02 on average after the escalation of the COVID pandemic, our estimation result implies that importing from China is an important channel through which the escalation of the COVID-19 pandemic increased firms’ subjective uncertainty. This effect is also large compared with the average firm-level uncertainty (CV) of 0.044 in the full sample. In columns (2) and (3) of Table 8, we examine other potential channels by utilizing the triple-interaction terms with firms’ export exposure and FDI exposure to China, respectively. It is found that the effects of the escalation of the COVID-19 pandemic on firms’ subjective uncertainty through exporting to China and FDI in China are weak and insignificant. The results in columns (4)–(6) using an alternative timing of the escalation of the COVID-19 pandemic are similar to the results in columns (1)–(3). Overall, our results show that the sudden escalation of the COVID-19 pandemic increased firm-level subjective uncertainty, especially through firms’ exposure to Chinese imports. These results suggest that the disruption of supply chains due to the outbreak of the COVID-19 pandemic played an important role in shaping the increasing uncertainty firms faced in January–February/2020.

**Table 8:** Import, export, and FDI exposure to China

	(1)	(2)	(3)	(4)	(5)	(6)
	$$\mathrm{Sales}_\mathrm{cv}$$					
$1(\mathrm{year}=2020)$	− 0.001 (0.005)	− 0.003 (0.005)	− 0.003 (0.005)	− 0.002 (0.004)	− 0.004 (0.004)	− 0.004 (0.004)
$1(\mathrm{year}=2020)\times 1(\mathrm{date}>\mathrm{Jan.}/26)$	0.004 (0.005)	0.008$^{+}$ (0.005)	0.005 (0.005)			
$1(\mathrm{year}=2020)\times 1(\mathrm{date}>\mathrm{Jan.}/22)$				0.004 (0.004)	0.007* (0.004)	0.005^+^ (0.004)
$1(\mathrm{year}=2020)\times \mathrm{Import}$	− 0.018 (0.024)			− 0.018 (0.024)		
$1(\mathrm{year}=2020)\times \mathrm{Export}$		0.079 (0.069)			0.080 (0.069)	
$1(\mathrm{year}=2020)\times \mathrm{FDI}$			0.004 (0.005)			0.004 (0.005)
$1(\mathrm{year}=2020)\times 1(\mathrm{date}>\mathrm{Jan.}/26)\times \mathrm{Import}$	0.069** (0.034)					
$1(\mathrm{year}=2020)\times 1(\mathrm{date}>\mathrm{Jan.}/26)\times \mathrm{Export}$		− 0.093 (0.112)				
$1(\mathrm{year}=2020)\times 1(\mathrm{date}>\mathrm{Jan.}/26)\times \mathrm{FDI}$			0.014$^{+}$ (0.011)			
$1(\mathrm{year}=2020)\times 1(\mathrm{date}>\mathrm{Jan.}/22)\times \mathrm{Import}$				0.067** (0.032)		
$1(\mathrm{year}=2020)\times 1(\mathrm{date}>\mathrm{Jan.}/22)\times \mathrm{Export}$					– 0.090 (0.111)	
$1(\mathrm{year}=2020)\times 1(\mathrm{date}>\mathrm{Jan.}/22) \times \mathrm{FDI}$						0.007 (0.010)
log(firm age)	− 0.041 (0.051)	− 0.033 (0.050)	− 0.027 (0.050)	− 0.030 (0.046)	− 0.026 (0.046)	− 0.021 (0.046)
Firm FE	Yes	Yes	Yes	Yes	Yes	Yes
$N$	636	636	636	724	724	724
$R^{2}$	0.679	0.674	0.678	0.683	0.679	0.680

Notes: $\mathrm{Sales}_\mathrm{cv}$ is the coefficient of variation of the (five-bin) expected sales distribution. Standard errors are clustered at the firm level. Dependent variable is trimmed out from both below and above at $1\%$ level. Firms that answered our survey between Jan./20/2020 and Jan./26/2020 are excluded from columns 1–3. Firms that answered our survey between Jan./20/2020 and Jan./22/2020 are excluded from columns 4–6. Significance levels: $^+$0.20, *0.10, **0.05, ***0.01

## Conclusion

Based on our original firm survey that contains five-bin forecasts for micro-level and macro-level variables, we measure firm-level expectations and uncertainty. We find that firm-level subjective uncertainty is statistically positively associated with a firm’s sales volatility. Taking advantage of the outbreak of COVID-19 in China in late January 2020, we find that the exogenous shock led to an increase in firm-level uncertainty, especially for firms doing business with China. In this way, based upon an event in which an unexpected shock occurred during the process of conducting the survey, our study analyzed the sort of impact that the shock had on firms’ future outlook. We found that, when confronted with a shock, there was an increase in variance in outlook prior to firms’ expected sales outlook being revised downward. Thus, the results confirmed the presence of uncertainty prior to firms tending toward pessimism; this is new knowledge obtained from this firm survey and will likely be also beneficial for policymakers in charge of the current economic situation as well as the outlook. This information has been used, for example, by the Bank of England, in its DMP surveys of variance in firms’ sales forecasts to prepare uncertainty metrics as well as to prepare documents for policymaker meetings and economic outlooks. The Federal Reserve Bank of Atlanta also conducts similar surveys. If Japan began to collect data about the uncertainty that firms face, this information might similarly be beneficial for policy and research.

## A Appendix

See Table 9.

**Table 9:** Summary statistics for regressions

	Count	Mean	SD	p5	p25	p50	p75	p95
Expected sales (log)	1115	8.278	1.158	6.390	7.512	8.274	8.997	10.266
Sales uncertainty (CV)	1113	0.046	0.034	0.009	0.022	0.036	0.059	0.110
Sales uncertainty (SD)	1091	0.045	0.033	0.009	0.021	0.037	0.059	0.110
China	1144	0.295	0.456	0.000	0.000	0.000	1.000	1.000
$1(\mathrm{year}=2020)$	1144	0.490	0.500	0.000	0.000	0.000	1.000	1.000
$1(\mathrm{year}=2020)\times 1(\mathrm{date}>\mathrm{Jan.}/26)$	806	0.239	0.427	0.000	0.000	0.000	0.000	1.000
$1(\mathrm{year}=2020)\times 1(\mathrm{date}>\mathrm{Jan.}/22)$	910	0.269	0.444	0.000	0.000	0.000	1.000	1.000
Import exposure	1144	0.034	0.108	0.000	0.000	0.000	0.000	0.308
Export exposure	1144	0.009	0.033	0.000	0.000	0.000	0.000	0.047
FDI exposure	1144	0.111	0.462	0.000	0.000	0.000	0.000	1.000

Notes: China is a dummy variable that equals one if the firm has (1) imports from China or, (2) exports to China, or (3) at least one manufacturing affiliate in China, and zero otherwise. Import exposure is the share of imports from China in total sourcing. Export exposure is the share of exports to China in total sales. FDI exposure is the number of manufacturing affilaites in China. We use the average values of import exposure, export exposure, and FDI exposure during the period 2013–2016 in the Kikatsu data

## Footnotes

1 We obtained financial support from JSPS KAKENHI (grant number 17H02531) and RIETI to run the survey. The first survey was executed by Tokyo Shoko Research (TSR) in October and November of 2017 and the second survey was executed by Teikoku Databank (TDB) in January–February 2020.

2 The validation tests for other variables yield qualitatively the same results.

3 Although 2185 firms responded to the survey in 2017, not all firms provided answers to all the questions. Therefore, the totals of firms that appear in a given row in Table 2 do not necessarily sum to 2185.

4 In the questionnaire, we ask the firm to report their forecasts in a weakly increasing order.

5 In the questionnaire, we ask the firm to report different forecasts in different bins.

6 For instance, we scale up or down the probabilities of the five bins uniformly when the total probabilities do not add up to one.

7 We obtain information of past sales from the Kikatsu dataset, as the two datasets share the same firm ID.

8 Although the difference seems visually small, the KS test confirmed that the distributions of sales uncertainty in 2017 and 2020 are not equal, with statistical significance at the $1\%$ level.

9 Source: https://www.worldometers.info/coronavirus/ [accessed March 13, 2021].

10 The number of confirmed cases was 53 on February 15, 2020 according to https://www.worldometers.info/coronavirus/country/japan/. Almost all firms answered the survey before February 15, 2020.

11 As the real-time data on imports/exports/overseas production are not available, it is not possible to further identify the demand/supply shocks, if any, during our sample period.

12 As we have firm fixed effects in the regression, we cannot insert the indicator variable that the firm answered the survey after January 26 or January 22, 2020, and its interaction term with the China dummy variable.

13 The remaining $38\%$ answered online.

14 730 firms answered our survey in both years, among which roughly 250 firms did not report the distribution of their forecasts (i.e., only reporting four or three bins or not reporting forecasts at all) and/or realized sales in either of the 2 years (i.e., 2017 and 2020); we exclude those firms from the regressions as expectations-related measures cannot be constructed for both years. Among the remaining (roughly) 480 firms, around 100 and 140 firms completed our survey in the periods January 20–22 and January 20–26. As a result, the base sample used in the various regressions contains 380 and 340 firms (i.e., 760 and 680 observations). However, since only observations that have nonmissing values for all the independent variables and the dependent variable can be included into the regressions (i.e., firm age, export to/imports from China, and the number of affiliates in China), we end up with around 730 and 640 observations in our various regressions.

15 One point worth noting is that although this table shows that roughly 1100 observations have nonmissing values for expected sales and sales uncertainty, this is not the maximum number of observations we can use in the regressions (without excluding firms that answered our survey in January 20–22 or January 20–26), as panel regressions in our paper require nonmissing values for expectations-related measures for both years. As noted, only 480 firms (or 960 observations) have nonmissing expectations-related measures for both years.

16 Our data show clearly that larger firms were more likely to answer the survey later than small firms. As a result, firms that answered our survey after January 26 (or January 22) were on average larger than those that answered the survey before January 26 (or January 22). The double-interaction term of the *China* dummy variable and whether the firm answered the survey after January 26 (or January 22) is absorbed by the firm fixed effects, as our measure of the exposure to the Chinese economy is time invariant.

17 For this information, we use the average value of (1) imports from non-China countries, (2) exports to non-China countries, and (3) the number of manufacturing affiliates in non-China countries during the period 2013–2016 in the Kikatsu data.
